# Co-trimoxazole versus azithromycin for the treatment of undifferentiated febrile illness in Nepal: study protocol for a randomized controlled trial

**DOI:** 10.1186/s13063-017-2199-6

**Published:** 2017-10-02

**Authors:** Sunil Pokharel, Buddha Basnyat, Amit Arjyal, Saruna Pathak Mahat, Raj Kumar KC, Abhusani Bhuju, Buddhi Poudyal, Evelyne Kestelyn, Ritu Shrestha, Dung Nguyen Thi Phuong, Rajkumar Thapa, Manan Karki, Sabina Dongol, Abhilasha Karkey, Marcel Wolbers, Stephen Baker, Guy Thwaites

**Affiliations:** 1 Oxford University Clinical Research Unit Nepal, Patan Academy of Health Sciences, Lalitpur, Nepal; 2 Centre for Tropical Medicine and Global Health, University of Oxford, Oxford, UK; 3 Patan Hospital, Patan Academy of Health Sciences, Lalitpur, Nepal; 4 Oxford University Clinical Research Unit, Ho Chi Minh City, Vietnam

**Keywords:** Undifferentiated febrile illness, Enteric fever, Azithromycin, Co-trimoxazole, Fever clearance time

## Abstract

**Background:**

Undifferentiated febrile illness (UFI) includes typhoid and typhus fevers and generally designates fever without any localizing signs. UFI is a great therapeutic challenge in countries like Nepal because of the lack of available point-of-care, rapid diagnostic tests. Often patients are empirically treated as presumed enteric fever. Due to the development of high-level resistance to traditionally used fluoroquinolones against enteric fever, azithromycin is now commonly used to treat enteric fever/UFI. The re-emergence of susceptibility of *Salmonella typhi* to co-trimoxazole makes it a promising oral treatment for UFIs in general. We present a protocol of a randomized controlled trial of azithromycin versus co-trimoxazole for the treatment of UFI.

**Methods/design:**

This is a parallel-group, double-blind, 1:1, randomized controlled trial of co-trimoxazole versus azithromycin for the treatment of UFI in Nepal. Participants will be patients aged 2 to 65 years, presenting with fever without clear focus for at least 4 days, complying with other study criteria and willing to provide written informed consent. Patients will be randomized either to azithromycin 20 mg/kg/day (maximum 1000 mg/day) in a single daily dose and an identical placebo or co-trimoxazole 60 mg/kg/day (maximum 3000 mg/day) in two divided doses for 7 days. Patients will be followed up with twice-daily telephone calls for 7 days or for at least 48 h after they become afebrile, whichever is later; by home visits on days 2 and 4 of treatment; and by hospital visits on days 7, 14, 28 and 63. The endpoints will be fever clearance time, treatment failure, time to treatment failure, and adverse events. The estimated sample size is 330. The primary analysis population will be all the randomized population and subanalysis will be repeated on patients with blood culture-confirmed enteric fever and culture-negative patients.

**Discussion:**

Both azithromycin and co-trimoxazole are available in Nepal and are extensively used in the treatment of UFI. Therefore, it is important to know the better orally administered antimicrobial to treat enteric fever and other UFIs especially against the background of fluoroquinolone-resistant enteric fever.

**Trial registration:**

ClinicalTrials.gov, ID: NCT02773407. Registered on 5 May 2016.

**Electronic supplementary material:**

The online version of this article (doi:10.1186/s13063-017-2199-6) contains supplementary material, which is available to authorized users.

## Background

The difficulty in determining the etiology of undifferentiated febrile illness (UFI), which designates fever without localizing signs, and the emergence of resistance to commonly used antimicrobials means that the appropriate management of UFIs is an ongoing clinical challenge. The non-specific clinical presentation of many infections that cause UFI makes it difficult to distinguish one cause from another based on clinical history, physical examination and basic initial laboratory investigations alone [1].

UFIs in industrialized countries are often attributed to a viral syndrome but in the developing world UFI may be commonly caused by enteric fever, rickettsial illness, malaria, dengue, chikungunya, etc. depending upon local epidemiology. The common causes of UFI in Kathmandu, Nepal includes enteric (typhoid and paratyphoid) fever and murine and scrub typhus [2, 3]. The pooled data of 2092 patients with UFI enrolled in four clinical trials conducted in the same study setting showed culture positivity in 885 (41%) patients for either *Salmonella typhi* or *Salmonella paratyphi* [4]. A study conducted in serum samples of a proportion of randomly selected patients from one of these trials showed serological evidence for acute murine typhus in 17% (*n* = 21/125) of patients [3]. Additionally, the spotted fever group rickettsioses, Q fever, leptospirosis, hantavirus infection, brucellosis and dengue may also cause UFI in Nepal [3].

There is no reliable point-of-care diagnostic test to determine rickettsial infections, self-remitting viral infections and many other febrile illnesses. Blood culture which is the “gold standard” test to determine enteric fever takes from 3 to 7 days. Therefore, treatment has to be guided by the clinical characteristics of the syndrome that the patient presents with and knowledge of local epidemiology of these common diseases. We have observed that most patients with UFI in Nepal are empirically treated as presumed enteric fever (in a high-incidence area) or as fever without a focus requiring antimicrobials (in a relatively low-incidence area). Our working definition for UFI is fever of ≥ 38.0 °C for at least 4 days’ duration with no clear-cut focus of infection such as productive cough, diarrhea, urinary symptoms, or any signs of neck stiffness or localized abscess. We exclude the patients with a fever duration of less than 4 days and a recorded temperature < 38 °C in an attempt to exclude the majority of patients with probable self-remitting viral infection. An appropriate antimicrobial, in addition to the supportive treatment, needs to be administered empirically to these patients in a setting where a large proportion of these UFIs is known to be caused by bacterial and rickettsial illnesses. Orally administered azithromycin is commonly used to treat UFI in Nepal and remains effective against enteric fever [5–7].

Fluoroquinolones cannot now be used to treat enteric fever in Nepal and South Asia as high levels of resistance have emerged against them within the *S. typhi* population [8–10]. This is further supported by endemic prevalence of a fluoroquinolone-resistant subclade of H58 *S. typhi* in Nepal [11]. This highlights the need for alternative antimicrobial treatments and to consider using the first-line drugs chloramphenicol, amoxicillin and co-trimoxazole for enteric fever [12]. These drugs are the “low-hanging fruits” in UFI treatment as suggested by by O’Neill’s [13] recent review on the global burden of antimicrobial resistance.

Co-trimoxazole has been commonly used in the past for the treatment of enteric fever [14, 15]. However, resistance to co-trimoxazole emerged two decades ago, but has subsequently largely disappeared and nearly all *S. typhi a*nd *S. paratyphi* A isolates from Nepal are now susceptible to this drug [9, 16–18]. Anecdotal reports claim that it performs well clinically for treating UFI in Nepal and it is a widely available at low cost. Its effectiveness against enteric fever is corroborated by a recent case report [19] and demonstrable low co-trimoxazole Minimum Inhibitory Concentrations (MICs) for *S. typhi* and *S. paratyphi* A [17, 20].

Both azithromycin and co-trimoxazole are available in Nepal and are extensively used in the treatment of UFI. Therefore, it is important to know the better oral option to treat enteric fever and other febrile illnesses. This would also aid in securing an alternative oral treatment in case resistance to azithromycin emerges.

## Methods/design

### Purpose/hypothesis

We hypothesize that azithromycin has a wider spectrum of activity against the major causes of UFI in Nepal (*S. typhi* and *S. paratyphi*; scrub and murine typhus) than co-trimoxazole and will, therefore, be associated with more rapid fever clearance times (FCTs) and less treatment failure.

### Objectives

#### Primary objective

To determine whether azithromycin has a faster FCT than co-trimoxazole for the treatment of UFI in Nepal.

#### Secondary objectives

- To determine whether azithromycin is superior to co-trimoxazole for the treatment of blood-culture-negative UFI
- To determine whether azithromycin is similarly efficacious as co-trimoxazole for the treatment of blood-culture-positive enteric fever
- To assess the causes of UFI in blood-culture-negative patients

### Study design

This is a parallel-group, double-blind, 1:1, randomized controlled phase-III trial of azithromycin versus co-trimoxazole for the treatment of UFI in Nepal.

### Study setting

The study will be conducted at Patan Hospital, the teaching hospital of Patan Academy of Health Sciences (PAHS), Lalitpur, Nepal. Patan Hospital is a tertiary-care center located in urban Lalitpur, with a large number of patients visiting the hospital for clinical care from both rural and urban areas of Kathmandu Valley and also a large number of patients referred from different parts of Nepal.

### Participants, interventions and outcomes

**Fig. 1:**
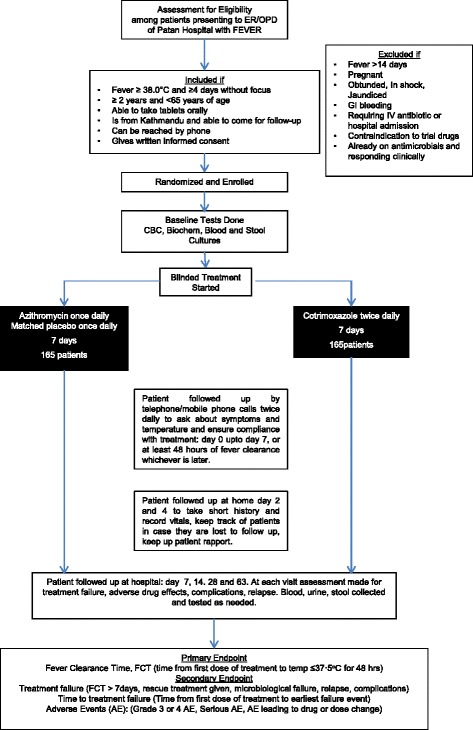
Descriptive flow chart of study plan

Participants will be the patients presenting with febrile illness to the emergency room and outpatient clinics of Patan Hospital and they will be recruited from the Fever Study Clinic at the hospital. The flow chart below illustrates the study plan for recruitment, intervention, outcome and follow-ups (see Fig. 1).

### Eligibility criteria

#### Inclusion criteria

1. Fever of ≥ 38.0 °C and for at least 4 days without a clear-cut focus of infection (as assessed by history and physical examination)
2. Older than 2 years and below 65 years of age
3. Able to take tablets orally
4. Residing in Kathmandu Valley
5. Able to attend follow-up visits
6. Can be contacted by telephone/mobile phone 24 h a day
7. Written informed consent to participate in the study including assent for minors in addition to parental consent

#### Exclusion criteria

1. Fever for more than 14 days
2. Pregnancy
3. Obtundation
4. Shock
5. Visible jaundice
6. Presence of signs of gastrointestinal bleeding
7. History of hypersensitivity to either of the trial drugs
8. Patient requiring intravenously administered antimicrobials or hospital admission for any reason as decided by the study physician and the attending physician
9. The study physician considers either of the trial drugs to be contraindicated for any reason (e.g., drug interactions, as described below)
10. Any patient fulfilling the inclusion criteria but already taking antimicrobials and responding clinically to the treatment as judged by the study physician

### Interventions

Patient will be randomized to one of the two treatment groups:

- Group A: azithromycin tablets 20 mg/kg/day as a single daily dose for 7 days (maximum dose 1000 mg/day)
- Group B: co-trimoxazole tablets (trimethoprim 10 mg/kg + sulfamethoxazole 50 mg/kg) in two divided doses everyday for 7 days (maximum 3000 mg/day)

The drug doses will be adjusted according to the weight of individual patients. An additional Excel sheet illustrates this in more detail (see Additional file 1)

### Description of study interventions

#### Azithromycin

Azithromycin is a macrolide antimicrobial derived from erythromycin, but has a methyl-substituted nitrogen in its 15-member lactone ring. It is, therefore, an azalide antimicrobial. It acts by inhibiting ribonucleic acid (RNA)-dependent protein synthesis by binding to the receptor at the bacterial 50S ribosomal subunit [21]. Azithromycin is available in capsules for oral use as azithromycin dihydrate, in film-coated tablets, as a powder for oral suspension and as a powder to prepare an intravenous solution.

#### Co-trimoxazole

Co-trimoxazole is a combination of two antimicrobial agents, trimethoprim and sulfamethoxazole, in the ratio 1:5.

Sulfamethoxazole is a sulfonamide, a structural analogue of para-aminobenzoic acid (PABA). It competes with PABA to bind to dihydropteroate synthetase and inhibit the conversion of PABA and dihydropteroate diphosphate to dihydrofolic acid, or dihydrofolate. This action inhibits the production of dihydrofolate intermediates and interferes with the normal bacterial synthesis of folic acid (folate) and deoxyribonucleic acid (DNA) synthesis [22].

Trimethoprim is a 2, 4-diamino-5-(3′,4′,5′-trimethoxybenzyl) pyrimidine. It has powerful inhibition activity on bacterial dihydrofolate reductase, which is the enzymatic step after the step in folic acid synthesis that is blocked by sulfonamides [23]. It thus interferes with the conversion of dihydrofolate to tetrahydrofolate, the precursor of folinic acid and ultimately of purine and DNA synthesis. The sequential blockage of the same biosynthetic pathway by sulfamethoxazole and trimethoprim results in a high degree of synergistic activity against a wide spectrum of microorganisms [23].

Co-trimoxazole is available as oral tablets, oral capsules and an oral suspension in different strengths combining trimethoprim and sulfamethoxazole in the ratio 1:5.

### Concomitant medications

The following medications should be avoided for patients in each treatment group:

#### Azithromycin

- Aluminum and magnesium antacids delay absorption and reduce the peak serum concentration and should be avoided with azithromycin

#### Co-trimoxazole

- Angiotensin-converting enzyme (ACE) inhibitors and angiotensin-receptor blockers have an additive hyperkalemic effect when used with co-trimoxazole
- The hypoglycemic action of sulfonylureas and thiazolidinediones is enhanced when concomitantly used with co-trimoxazole
- Co-trimoxazole interacts with warfarin through the CYP450 and CYP2C9 systems, increasing the risk of bleeding
- Concomitant administration of co-trimoxazole and methotrexate results in decreased renal clearance of free methotrexate and may result in severe pancytopenia
- Active levels of phenytoin may be increased markedly by co-trimoxazole, especially in older patients
- Concomitant use may increase the serum level of other drugs like rifampicin, dapsone, amiodarone, digitalis, amantadine, pyrimethamine, cyclosporine, fluoxetine, montelucast, zafirlukast, paclitaxel

### Outcomes

#### Primary endpoints

Fever clearance time (FCT): this will be the time from the first dose of a study drug until a temperature of ≤ 37 · 5 °C has been achieved for at least 48 h.

#### Secondary endpoints

1. Treatment failure, defined as the occurrence of at least one of the following events
  - Fever failure, defined by FCT above 7 days (168 h) post treatment initiation
  - Requirement for rescue treatment as judged by the study physician and the attending physician
  - Blood-culture-0positive for *S. typhi* or an *S. paratyphi* on day 7 of treatment regardless of fever (microbiological failure)
  - Culture-confirmed or syndromic enteric fever relapse within 28 days of initiation of treatment
  - The development of any complication (e.g., clinically significant bleeding, fall in the Glasgow Coma Scale score, perforation of the gastrointestinal tract) or need for hospital admission within 28 days after the initiation of treatment
2. Time to treatment failure, defined as the time from the first dose of treatment until the date of the earliest failure event
3. Adverse events (grade-3/4/5 adverse events, serious adverse events, adverse events of any grade leading to modification of study-drug dose or interruption/early discontinuation)

### Rescue treatment and post-trial treatment

Patients who meet the criteria for treatment failure as defined above will be administered rescue treatment. Rescue treatment will be as follows: treatment failures with a positive blood culture for *S. typhi* and *S. paratyphi* at the time of enrollment will be treated with injected ceftriaxone 60 mg/kg once daily (maximum dose 2 g/day) for 7 days and culture-negative individuals will be treated with injected ceftriaxone 60 mg/kg once daily (maximum dose 2 g/day) and doxycycline tablet 4 mg/kg/day in two divided doses (maximum 200 mg/day) for 7 days.

Patients who develop adverse events (grade-3/4/5 adverse events, serious adverse events, adverse events of any grade leading to modification of study drug dose or interruption/early discontinuation) will stop receiving the study drug and be given rescue treatment as above.

If treatment failure occurs in patients who are found to be infected with organisms other than *S. typhi* or *S. paratyphi* the choice of the rescue treatment will be determined after full clinical assessment and consideration of the laboratory results by the study physician in consultation with the attending physician.

Patients requiring hospital admission for any reason, as decided by the study physician and the attending physician, will be admitted to the hospital and treated as per standard clinical care. They will remain in the study with all outcomes recorded. Patients will be treated according to standard clinical care for all adverse events and upon completion of the study as required.

### Participant timeline

#### Identification of participants, consent and enrollment

Patients aged 2 years and older and under 65 years who present at the emergency room and outpatient clinics of Patan Hospital, who have a temperature of ≥ 38.0 °C and a documented or self-reported history of fever for at least 4 days and less than 14 days, without a focus of infection (as assessed by history and physical examination), will be identified by clinicians in the hospital and will be referred to the study clinic. At the study clinic, the study physician will screen the patients by study criteria for enrollment into the study.

The study physician will obtain written informed consent from all the study participants aged over 18 years of age before enrollment in the study. For patients aged 12 to 18 years of age, written informed consent will be obtained from a legal guardian in addition to an assent from the participant. Written informed consent will be obtained from legal guardians for patients under 12 years of age. In case of legal guardians/patients who cannot read, an impartial witness, who is not a part of the research team (if the patient is not accompanied by someone who is literate, this can be one of the staff members from the hospital medical record office), will sign to confirm that the Informed Consent Form was accurately read to the patient and that the patient agreed to participate. The study physician will also approach participants/legal guardians for an additional consent for storage of blood and urine samples for future studies. All study participants will receive a copy of the signed Informed Consent Form.

After signing an informed consent, patient will be assigned a study number (this is the same as the randomization number) and will be enrolled into the study. The study staff will ensure that the participants’ anonymity is maintained. Participants will be identified only by their initials and a study number. All documents will be stored securely and will be accessible to trial staff and authorized personnel only.

### Screening failure

Patients who do not meet the inclusion/exclusion criteria for the study will be informed that they cannot participate in the trial and will be treated according to the standard protocol of clinical care including antimicrobial therapy. Details of all patients who are approached for participation in the trial will be noted in the “Screening Case Report Form (CRF).”

### Baseline procedures

After signing the informed consent, but prior to enrollment, patients will undergo:

- Full history and clinical examination including documentation of clinical manifestations according to a standard proforma
- Complete blood count (CBC): hematocrit, total white cell count, differential count, platelet count (blood volume 1 ml)
- Biochemistry: blood glucose, creatinine, serum glutamine oxaloacetate transaminase (SGOT), serum glutamine pyruvate transaminase (SGPT) (blood volume 1 ml)
- Urine for routine examination and storage
- Blood culture: blood volume 5–8 ml for patients aged at least 14 years of age and 3 ml for patients below 14 years of age
- EDTA-preserved blood will be separated into plasma and a cell pellet and stored for future testing (3 ml)
- Stool culture: collect in readymade tubes with Cary Blair medium

The causes of fever will be investigated in all patients according to routine clinical practice norms. Patients will be screened by other investigations for any specific diseases prior to enrollment in case of strong clinical suspicion during history taking and clinical examination; for example, a chest X-ray for patients with cough and/or pleuritic chest pain to rule out lower respiratory tract infections, urine routine examination for urinary symptoms, CBC to screen for leukocytosis if any symptom are suggestive of a specific focus of infection, etc.

### Study duration

**Fig. 2:**
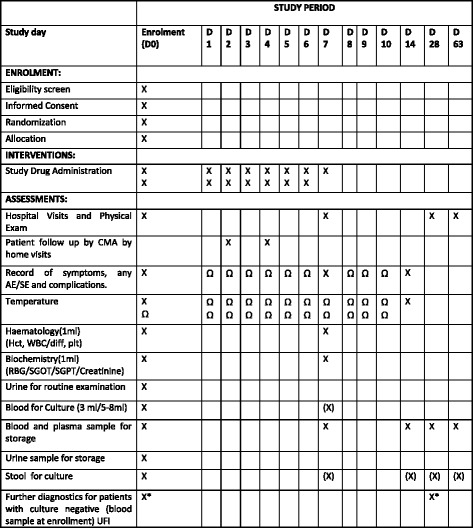
Standard Protocol Items: Recommendations for Interventional Trials (SPIRIT) Figure: summary of enrollment, intervention and assessment. *X* Activities at hospital or home visits, *Ω* Activities that will be recorded over a telephone call, *(X)* Will be done only for culture-positive patients at day 0 or those with persistent fever/symptoms, X* Will be done retrospectively if blood culture shows no growth after 7 days

For each enrolled patient, study duration will be from day 0 (day of enrollment) until day 63 (day of final follow-up). The schedule for intervention and assessment during the study period is illustrated in a Standard Protocol Items: Recommendation for Interventional Trials (SPIRIT) Figure, see Fig. 2.

### Day 0 – day 7

Patients will be contacted by telephone calls twice daily by study nurses between 06:30 and 07:30 and 18:30 and 19:30 to record drug doses and administration times, oral temperatures and time of temperature readings, symptoms and potential adverse effects.

Each patient will be given a digital thermometer and the “Patient Copy of Temperature and Medication Record” page of the CRF and will be asked to record their oral temperature and amount of paracetamol intake at 06:00 and 18:00 and during any febrile episode between each day; and indicate study-drug intake with a tick mark. “Hospital Copy of Temperature and Medication Record” will be verified with the “Patient Copy of Temperature and Medication Record” when patients follow-up on day 7. A symptom checklist will also be completed daily and recorded to assess complications or adverse events.

Patient will be followed up by home visits by community medical auxiliaries (CMAs) on day 2 and day 4 to monitor patients’ vital signs, keep track of patients in case they are lost to follow-up and maintain patient rapport.

### Follow-up: day 7 (±1 day)

Patients will be examined by the study physician at study clinic. The following tests will be performed:

- Blood will be collected in a 10-ml syringe for CBC, random blood glucose (RBG), SGPT, SGOT, creatinine, culture and plasma storage for patients whose blood culture is positive for *S. typhi* or *S. paratyphi* A at the day of enrollment
- Blood will be collected in a 5-ml syringe for CBC, RBG, GPT, GOT, creatinine and plasma storage for patients who are blood-culture-negative on the day of enrollment. If culture-negative patients have not cleared their fever on day 7, a blood culture will also be performed
- Stool culture (collected in readymade tubes with Cary Blair medium) for patients whose blood is culture-positive for *S. typhi* or *S. paratyphi* A on the day of enrollment

### Day 8 – day 10

For the patients who are febrile on days 6/7, daily telephone calls and a record of oral temperature and symptoms will be continued in the same manner.

### Follow-ups: day 14 (±2 days), day 28 (±3 days), day 63 (±3 days)

Patients will be reexamined by the study physician at the study clinic. The following tests will be performed:

- Blood will be collected in a 3-ml syringe for plasma storage
- Stool culture (collected in readymade tubes with Cary Blair medium) for patients whose blood culture is positive for *S. typhi* or *S. paratyphi* A on the day of enrollment

### Further diagnostic tests

Further diagnostic tests for UFI, including enzyme-linked immunosorbent assay (ELISA) and polymerase chain reaction (PCR), will be performed on archived paired samples of blood collected on days 1 and 28 ± 3 days for scrub typhus, murine typhus, spotted fever group rickettsioses, Q fever, leptospirosis, hantavirus infection, brucellosis and dengue on patients whose initial blood culture shows no growth for *S. typhi* or *S. paratyhi*. These tests will be conducted in the future to determine the causes of UFI and will not influence present clinical care. It will be explained to patients that if any additional tests performed on their blood samples are positive, they will be informed once the results are available.

### Discontinuation of treatment and participation

If a patient or patient representative, who has given consent on their behalf, opts to discontinue the trial treatment for any reason, they are encouraged to follow the study procedures and attend follow-ups as far as possible instead of completely withdrawing from the trial. If they do not wish to remain on trial follow-up, however, their decision will be respected and the patients will be completely withdrawn from the trial. The reason for the patient withdrawing should be ascertained wherever possible. Prior to withdrawing from the trial, the patient will be invited to have assessments performed as appropriate for the final visit although they would be at liberty to refuse any or all individual components of the assessment.

In addition, the investigator may decide to stop the study intervention if they feel that it is not in the best interest of the patient. Patients will be followed as per protocol.

### Sample size

The target sample size for this trial is 330 participants (165 per study group). This sample size is based on the sample size justification for 300 patients with an additional allowance of 10% to account for potential loss to follow-up and other deviations from the protocol assumptions.

### Sample size justification

We assumed a Weibull distribution for the FCT in each arm and a median FCT in the azithromycin arm of 1.12 days in those with culture-negative UFI and 2.78 days in those with culture-positive UFI. This corresponds to the observed values for gatifloxacin in culture-negative patients and ceftriaxone for culture-positive patients in our previous trial [9]. The shape parameter of the Weibull distribution was assumed to be 0.75 for culture-negative patients and 1.5 in culture-positive patients. This estimation is conservative (i.e., implies slightly more variability) compared to the observed FCT distributions in the previous three typhoid trials conducted in Nepal [8, 20, 24]. Regarding the effect size, we assume that co-trimoxazole is associated with a two-fold slower FCT in culture-negative patients and the same FCT as azithromycin in culture-positive patients. Of note, for the culture-negative patients, a 2.26-fold slower FCT was observed in the ceftriaxone arm compared to gatifloxacin in our previous trial [9]. Finally, we assumed that the proportion of patients with culture-confirmed typhoid is between one third and one half of the total population. Based on these assumptions and an assumed twice-daily temperature monitoring leading to an interval-censored FCT, power for varying samples sizes was estimated based on simulation. Results for the chosen total sample size of *n* = 300 (150 per treatment group) are reported below (see Table 1) and provide a power of ≥ 80% for the overall comparison with a two-sided 5% significance level.

**Table 1:** Power for the overall population, culture-negative patients and culture-positive patients based on the assumptions above

Total sample size in both groups (culture-positive/-negative)	All patients (ITT): power for superiority of azithromycin	Culture-negative patients: power for superiority of azithromycin	Culture-positive patients: power for “non-inferiority” of azithromycin^a^
300 (150/150)	80%	89%	96%
300 (100/200)	92%	95%	86%

^a^Probability that the 95% confidence interval for the effect of azithromycin excludes the possibility that azithromycin is associated with a 1.5-fold slower fever clearance time (FCT) in culture-positive patients. *ITT* intention-to-treat

### Assignment of interventions

#### Randomization and blinding

All patients enrolled into the study will be randomly assigned in a 1:1 ratio to either co-trimoxazole or azithromycin according to a computer-generated randomization list, with randomization in variable block sizes of 4 and 6 without stratification. The randomization list will specify the assignment of each unique study number to the respective randomized treatment arm.

The study drugs have been manufactured by Lomus Pharmaceuticals, Kathmandu, Nepal. The tablets have been manufactured as follows: co-trimoxazole tablets of dose 1200 mg, 600 mg and 300 mg and 150 mg, azithromycin tablets of dose 800 mg, 400 mg, 200 mg and 100 mg, and placebo tablets in four different sizes, namely placebo tablet 1, placebo tablet 2, placebo tablet 3 and placebo tablet 4. The tablets have been manufactured such that the tablets mentioned in each column below are identical in shape and size and all the tablets have been coated (see Table 2).

**Table 2:** Doses formulation for equivalent tablets in two arms

Tablet 1	Tablet 2	Tablet 3	Tablet 4
Azithromycin 800-mg tablet	Azithromycin 400-mg tablet	Azithromycin 200-mg tablet	Azithromycin 100-mg tablet
Placebo tablet 1	Placebo tablet 2	Placebo tablet 3	Placebo tablet 4
Co-trimoxazole 1200-mg tablet	Co-trimoxazole 600-mg tablet	Co-trimoxazole 300-mg tablet	Co-trimoxazole 150-mg tablet

The central study pharmacist and an assistant will have access to the randomization list. They will not be otherwise involved in the conduct of the study in terms of seeing or enrolling patients or accessing the patients during follow-up and handling patient data. The drugs will prepackage and for each enrolled patient, a set of all the tablets of various sizes, sufficient for the entire course of study treatment, in two different, sealed and numbered envelopes for odd and even doses as per the randomization list, will be given.

At enrollment, the study staff will explain to the patient to take the appropriate tablets as per the drug-dosing table according to the weight of the patient. Refer to the additional Excel sheet for the details (see Additional file 2). The respective doses for each patient of a particular weight will be identical for both treatment groups.

Patients in the co-trimoxazole arm will receive both an odd and an even number of doses of co-trimoxazole tablets each day. Similarly, patients in the azithromycin arm will receive an odd number dose of azithromycin tablets and an even number dose of placebo tablets to blind disparity of drug frequency each day (see Table 3).

**Table 3:** Treatment allocations in each arm

Azithromycin arm		Co-trimoxazole arm	
Odd dose	Even dose	Odd dose	Even dose
Azithromycin tablets	Placebo tablets	Co-trimoxazole tablet	Co-trimoxazole tablet

- Odd number dose: doses 1, 3, 5, 7, 9, 11 and 13
- Even number dose: doses 2, 4, 6, 8, 10, 12 and 14

Treatment allocation will be concealed from the patient, investigators, study physician, study nurse and other study staffs throughout the study and will hence be double blinded.

### Administration and compliance

The first dose of the study drug will be administered under direct observation of the study physician in the hospital. If there is no hypersensitivity reaction, the patient will be discharged. Patients will be advised to take medication as per the study protocol and their compliance will be monitored by daily telephone calls and home visits on days 2 and 4.

To account for the need for re-administration in cases of vomiting, patients will be additionally given doses 15, 16, 17 and 18. If a patient has vomiting within 30 min of taking the drug, and if the volume is significant or the tablets are visible in the vomitus, the dose vomited should be re-administered from the same pouch. No dose will be re-administered more than once. If there are more than four episodes of vomiting after taking drugs, patients will be asked to visit the hospital and will be considered for discontinuation of the given study drug.

### Un-blinding

Un-blinding denotes revealing the identity of the study treatment. Un-blinding will take place in the event that the patient wishes to discontinue participation in the study or when the study physician and attending physician feel that the study drug needs to be un-blinded due to drug side effects. Study treatment should only be un-blinded if knowing the treatment will result in a change in the patient’s management.

The decision as to whether or not to un-blind should be discussed with the principal investigator (PI). Un-blinding will take place in the presence of the pharmacist and the attending physician. Un-blinding will be documented in the CRF.

### Data collection, management and analysis

#### Data handling and storage of records

Patient data will be recorded on paper CRFs, and entered on electronic CRFs later on a secure database via laptop computers, by study physicians and study nurses and transferred to the Data Management Team. Original paper study records will be kept for a minimum of 15 years in a secure location.

### Data access

The final trial dataset will be accessible to the trial team in the two collaborating institutions, OUCRU-Nepal at PAHS and OUCRU-Vietnam. All decisions on trial data use for analysis will be made by the PI. The data-entry team, which comprises study nurses and study physicians, will have access to the database forms. The IT team at OUCRU-Vietnam will have access to the database worksheets which they will provide to the DSMB statistician for interim analysis and to the study statistical analysis team for the end-of-study analysis. All electronic data will be kept safe and securely backed up for at least 15 years after the end of the study.

### Statistical analysis

The primary analysis population for all analyses is the full analysis population including all randomized subjects. Patients will be analyzed according to their randomized arm (intention-to-treat). In addition, the analyses will be repeated in the subsets of patients with blood-culture-confirmed enteric fever and in culture-negative patients.

The primary endpoint, the interval-censored FCT, will be compared between the two groups based on a Weibull accelerated-failure-time model with the treatment arm as the only covariate. The distribution of the FCT over time in each treatment arm will be further visualized using the non-parametric maximum likelihood estimator (NPMLE).

To summarize treatment failures, we will analyze both the time to treatment failure and the absolute risk of treatment failure until day 28. The former will be displayed with Kaplan-Meier curves and compared between the two groups with a Cox regression model with treatment as the only covariate. The Kaplan-Meier estimate on day 28 will be used as the estimate of the absolute risk of treatment failure and the comparison will be based on standard errors according to Greenwood’s formula.

Predefined subgroup analyses for both FCT and the time to treatment failure are by culture result (blood culture-positive or -negative), age (below 14 years or 14 years and older) and by pathogen in culture-confirmed patients (*S. typhi* or *S. paratyphi* A).

If further diagnostic tests, including ELISA and PCR, for the blood-culture-negative cases identify subsets of the study population with the same alternative diagnosis, including at least 15 subjects, outcomes in these subsets will also be analyzed.

This trial utilizes a pragmatic dosing regimen; that is, the tablets will be dispensed without breaking and a near accurate dose will be given in each treatment arm. To assess the effect of variation in the actual dose, a subgroup analysis will be conducted by the actual dose that a patient is given. This is derived from the intended dose from the patient’s body weight and drug-dosing table (see Additional file 1). This is a baseline measurement set at the time of randomization and will be known once un-blinding is done.

### Dissemination policy

We will publish the trial results in Hospital Grand Rounds and Medical Society meetings in Nepal. We will also use the Rotary Club and other such venues to disseminate the findings of the study as a way of public engagement. At the end of the study, the findings will be published on an open-access, peer-reviewed medical journal.

### Authorship eligibility guidelines

Authorship eligibility will be based on current recommendations of the International Committee of Medical Journal Editors (ICMJE). We are not using professional writers.

### Monitoring

#### Data and Safety Monitoring Board (DSMB)

An independent Data and Safety Monitoring Board will be set up which will consist of qualified volunteers with the necessary knowledge of clinical trials. The DSMB will review and approve a monitoring plan before the study commences. This will include how often the DSMB should receive summary reports. All data reviewed by the DSMB will be in the strictest confidence. A DSMB charter will outline its responsibilities and operational plan. The DSMB will be notified within 7 days of the PI being aware of the occurrence of a serious unexpected adverse event. An interim analysis to compare the primary outcomes in the two groups will be carried out once the data from the day-28 visit is available from the 100th and 200th patients and the report will be submitted to the DSMB.

The PI will also submit an annual progress report (or when requested) to the relevant Research Ethic Committees (Oxford University and Nepal Health Research Council), host organization and sponsor. In addition, an end-of-study notification and final report will be submitted to the same parties.

### Risks

Study procedures, including physical examination and phlebotomy, are standard clinical-care procedures and carry minimal risks. Phlebotomy may result in bruising, pain or infection at the site. The volume of blood drawn from children is below the WHO recommended limits for research in sick children [25].

The investigators will retain custody of the data and samples that will be jointly protected by the collaborating institutions according to established procedures and security barriers. Any future use of stored samples carries risk of loss of privacy and possibly stigmatization. The appropriate ethical committees will evaluate these and other issues before any testing is performed.

Treatments in both arms are commonly used in this setting with very few complications. Administration of the first dose of both will be in the hospital setting to reduce the risk associated with the possibility of allergic reaction.

### Side effects of azithromycin

- Common: diarrhea, nausea, abdominal pain
- Uncommon: palpitations, chest pain, jaundice, vomiting, dizziness, headache, vertigo, fatigue, inflammation and allergic reaction, decreased hearing, blurred vision, dark urine

### Side effects of co-trimoxazole

- Common: loss of appetite, nausea, vomiting, diarrhea, reversible exanthemas, headache, fever
- Uncommon: severe allergic skin reactions (Stevens-Johnson syndrome, Lyell’s syndrome, hematological complications (e.g., agranulocytosis, pancytopenia), cholestatic jaundice, pancreatitis, colitis, nephritis, vasculitis, aseptic meningitis, neuropsychiatric symptoms

Side effects will be monitored by telephone calls and the patients will be asked to attend to hospital when necessary.

### Adverse events and reporting

#### Definitions

An adverse event (AE) is any untoward medical event that occurs to a study participant during the course of the study whether or not that event is considered related to the study drug. An AE can, therefore, be any unfavorable and unintended sign, a symptom, or disease (including an abnormal laboratory finding) temporarily associated with the study drug, whether or not considered related to the study drug. Clinical or laboratory events are considered AEs only if they occur after the first dose of study treatment. Stable chronic conditions, such as arthritis, which are present prior to clinical trial entry and do not worsen are not considered AEs and will be documented in the subject’s clinical chart as medical history.

Serious adverse event (SAE) an AE is considered to be “serious” if it results in one of the following outcomes:

- Death
- Life-threatening event (the subject was at immediate risk of death at the time of the event; it does not refer to an event which hypothetically might have caused death if it were more severe)
- Inpatient hospitalization (new admissions) or prolongation of existing hospitalization (beyond what is expected for normal clinical care)
- Persistent or significant disability/incapacity (a substantial disruption of a person’s ability to conduct normal life functions)
- Congenital anomaly/birth defect

Patients will be followed up until the resolution of such symptoms even after the study comes to an end.

Any undiagnosed pregnancy during which treatment occurred will be followed until outcome. Any congenital abnormality or birth defect will be recorded as a SAE.

Unexpected serious adverse events (USAEs) are untoward medical events which fit one or more of the criteria for SAEs above and which are not considered a part of normal clinical progression of disease or an expected reaction to standard treatment therapy. Any event that becomes of concern to the investigators or study physicians during the course of the trial may be reported as a USAE.

### Reporting

All adverse events will be fully recorded in the “Adverse events page” in the CRF including duration, severity, outcome and relationship to the study drug. We will refer to the Common Terminology Criteria for Adverse Events (CTCAE V4). All adverse events will be noted in the study based on the Patient-reported Symptom Questionnaire and by probing questions based on the AE page of the CRF by the study physician on follow-up visits on days 7 and 14.

### Trial auditing

The trial will undergo both internal and external auditing. Internal auditing will be performed on a day-to-day basis by study physicians, study nurses and the Clinical Trial Unit, who will check data details and any discrepancies will be met with follow-up maintenance and appropriateness of patient recruitment. External auditing will be carried out by the Clinical Trials Unit at our collaborating research unit, OUCRU-VN, twice a year with reassessment of all study documents and CRFs and a review of crucial trial events.

## Discussion

There have been several published studies that used azithromycin or co-trimoxazole for the treatment of enteric fever, but there have been no head-to-head comparisons of the two drugs for UFI [5–7, 14, 15]. The studies that have used co-trimoxazole have generally been of small sample size and have not included all UFIs [14, 15].

Azithromycin is recommended for the treatment of the rickettsial fevers but its usefulness for UFI treatment in settings where rickettsial infections and enteric fever are common needs to be assessed [26]. There have been no trials performed for the treatment of UFI in our setting of fluoroquinolone-resistant enteric fever.

There have been attempts to develop protocols for the initial investigations and management of UFIs in South Asia [27], but the choice of antimicrobial in such protocols is still empirical and guided by subjective judgment. This study should pave the way for defining antibiotic guidelines for such presentations in Nepal.

The findings of this study will be important for clinicians as it will help them choose the best empirical treatment for UFI and reduce the rates of treatment failures and the risk of complications, and thus directly reduce patient suffering. This will also avert the need of using multiple antimicrobials to treat a common presentation of illness in Nepal and South Asia and thus may prevent the overuse of antimicrobials especially in the treatment of UFI.

This paper outlines the study protocol following the Standard Protocol Items: Recommendations for Interventional Trials (SPIRIT) guidelines for intervention trials (the SPIRIT Checklist is included as Additional file 3)

### Trial status

The trial is currently ongoing. It started enrollment in June 2016 and aims to be completed in June 2019. At the time of acceptance of this manuscript for publication, 90 participants (27% of the estimated sample size) have been enrolled into the study.

## Additional files

Additional file 1: Drug-dosing table. Calculation of drug dose according to the patients’ weight in each arm. (XLSX 21 kb)

Additional file 2: Simplified drug-dosing table. A simplified drug-dosing table derived from a drug-dosing table which explains the number of tablets required for each kilogram weight of the patient irrespective of the treatment arm. (XLSX 9 kb)

Additional file 3: SPIRIT Checklist. A checklist according to Standard Protocol Items: Recommendations for Interventional Trials (SPIRIT) guidelines. (DOCX 52 kb)

## Abbreviations

CBC: Complete blood count
CRF: Case Record Form
CMA: Community medical auxiliary – a research assistant who works full time on the study
CTCAE: Common Terminology Criteria for Adverse Events
FCT: Fever clearance time
OUCRU: Oxford University Clinical Research Unit
PAHS: Patan Academy of Health Sciences
RBG: Random blood sugar
SGOT: Serum glutamine oxaloacetate transaminase
SGPT: Serum glutamine pyruvate transaminase
UFI: Undifferentiated febrile Illness
USAE: Unexpected serious adverse event

## References

[1] Crump JA, Kirk MD (2015). Estimating the burden of febrile illnesses. PLoS Negl Trop Dis..

[2] Zimmerman MD, Murdoch DR, Rozmajzl PJ, Basnyat B, Woods CW, Richards AL (2008). Murine typhus and febrile illness, Nepal. Emerg Infect Dis.

[3] Thompson CN, Blacksell SD, Paris DH, Arjyal A, Karkey A, Dongol S (2015). Undifferentiated febrile illness in Kathmandu, Nepal. Am J Trop Med Hyg.

[4] Thompson CN, Karkey A, Dongol S, Arjyal A, Wolbers M, Darton T (2017). Treatment response in enteric fever in an era of increasing antimicrobial resistance: an individual patient data analysis of 2092 participants enrolled into 4 randomized, controlled trials in Nepal. Clin Infect Dis..

[5] Dolecek C, La TTP, Rang NN, Phuong LT, Vinh H, Tuan PQ (2008). A multi-center randomised controlled trial of gatifloxacin versus azithromycin for the treatment of uncomplicated typhoid fever in children and adults in Vietnam. PLoS One.

[6] Parry CM, Ho VA, Phuong LT, Bay PVB, Lanh MN, Tung LT (2007). Randomized controlled comparison of ofloxacin, azithromycin, and an ofloxacin-azithromycin combination for treatment of multidrug-resistant and nalidixic acid-resistant typhoid fever. Antimicrob Agents Chemother..

[7] Chinh NT, Parry CM, Ly NTHI, Ha HDUY, Thong MAIX, Diep TOS (2000). A randomized controlled comparison of azithromycin and ofloxacin for treatment of multidrug-resistant or nalidixic acid-resistant enteric fever. Antimicrob Agents Chemother..

[8] Koirala S, Basnyat B, Arjyal A, Shilpakar O, Shrestha K, Shrestha R (2013). Gatifloxacin versus ofloxacin for the treatment of uncomplicated enteric fever in Nepal: an open-label, randomized, controlled trial. PLoS Negl Trop Dis..

[9] Arjyal A, Basnyat B, Nhan HT, Koirala S, Giri A, Joshi N (2016). Gatifloxacin versus ceftriaxone for uncomplicated enteric fever in Nepal: an open-label, two-centre, randomised controlled trial. Lancet Infect Dis..

[10] Parry CM, Thuy CT, Dongol S, Karkey A, Vinh H, Chinh NT (2010). Suitable disk antimicrobial susceptibility breakpoints defining *Salmonella enterica serovar typhi* isolates with reduced susceptibility to fluoroquinolones. Antimicrob Agents Chemother..

[11] Thanh DP, Karkey A, Dongol S, Thi NH, Wong V, Tran N, et al. A novel ciprofloxacin-resistant subclade of H58 *Salmonella typhi* is associated with fluoroquinolone treatment failure. Elife. 2016;5:e14003. 10.7554/eLife.14003 PMC4805543 26974227

[12] Sharvani R, Dayanand DK, Shenoy P, Sarmah P (2016). Antibiogram of salmonella isolates : time to consider antibiotic salvage. J Clin Diagnostic Res..

[13] O’Neill J. Tackling drug-resistance infections globally: final report and recommendations. https://amrreview.org/sites/default/files/160518_Final%20paper_with%20cover.pdf.

[14] Thisyakorn U, Pethai M. Comparative efficacy of mecillinam, mecillinam/amoxicillin and trimethoprim-sulfamethoxazole for treatment of typhoid fever in children. Pediatr Infect Dis J. 1992;11:979–80. 1454448

[15] Schiraldi O, Sforza E, Piaia F (1985). Effect of a new sulfa-trimethoprim combination (trimethoprim-sulfamethopyrazine) in typhoid fever. A double-blind study on 72 adult patients. Chemotherapy.

[16] Shrestha KL, Pant ND, Bhandari R, Khatri S, Shrestha B. Re-emergence of the susceptibility of the *Salmonella* spp. isolated from blood samples to conventional first line antibiotics. Antimicrob Resist Infect Control. 2016;5:22. 10.1186/s13756-016-0121-8 PMC4881163 27231547

[17] Chand HJ, Rijal KR, Neupane B, Sharma VK, Jha B (2014). Re-emergence of susceptibility to conventional first line drugs in *Salmonella* isolates from enteric fever patients in Nepal. J Infect Dev Ctries..

[18] Maskey AP, Basnyat B, Thwaites GE, Campbell JI, Farrar JJ, Zimmerman MD (2008). Emerging trends in enteric fever in Nepal: 9124 cases confirmed by blood culture 1993–2003. Trans R Soc Trop Med Hyg..

[19] Karki M, Pandit S, Baker S, Basnyat B (2016). Cotrimoxazole treats fluoroquinolone-resistant *Salmonella typhi* H58 infection. BMJ Case Rep..

[20] Arjyal A, Basnyat B, Koirala S, Karkey A, Dongol S, Agrawaal KK (2011). Gatifloxacin versus chloramphenicol for uncomplicated enteric fever: an open-label, randomised, controlled trial. Lancet Infect Dis..

[21] Piscitelli S, Danziger LHRK (1992). Clarithromycin and azithromycin: new macrolide antibiotics. Clin Pharm..

[22] Smith L (1964). Evaluation of a new sulfonamide, sulfamethoxazole (Gantanol). JAMA.

[23] Bushby SR, Hitchings GH (1968). Trimethoprim, a sulphonamide potentiator. Br J Pharmacol Chemother.

[24] Pandit A, Arjyal A, Day JN, Paudyal B, Dangol S, Zimmerman MD (2007). An open randomized comparison of gatifloxacin versus cefixime for the treatment of uncomplicated enteric fever. PLoS One..

[25] Howie S (2011). Blood sample volumes in child health research : review of safe limits. Bull World Health Organ.

[26] Aung AK, Spelman DW, Murray RJ, Graves S (2014). Review article: rickettsial infections in Southeast Asia: implications for local populace and febrile returned travelers. Am J Trop Med Hyg..

[27] Thangarasu S, Natarajan P, Rajavelu P, Rajagopalan A (2011). A protocol for the emergency department management of acute undifferentiated febrile illness in India. Int J Emerg Med..

[28] Kong H, West S (2008). Ethical principles for medical research involving human subjects. World Med Assoc Declaration Helsinki..

